# Emergency Department Visits by Older Adults with Mental Illness in North Carolina

**DOI:** 10.5811/westjem.2015.8.27662

**Published:** 2015-11-16

**Authors:** Anne M. Hakenewerth, Judith E. Tintinalli, Anna E. Waller, Amy Ising

**Affiliations:** * North Carolina Department of Health and Human Services, Division of Public Health, Raleigh, North Carolina; † University of North Carolina at Chapel Hill, Department of Emergency Medicine, Chapel Hill, North Carolina; ‡ University of North Carolina at Chapel Hill, Carolina Center for Health Informatics, Department of Emergency Medicine, Chapel Hill, North Carolina

## Abstract

**Introduction:**

We analyzed emergency department (ED) visits by patients with mental health disorders (MHDs) in North Carolina from 2008–2010 to determine frequencies and characteristics of ED visits by older adults with MHDs.

**Methods:**

We extracted ED visit data from the North Carolina Disease Event Tracking and Epidemiologic Collection Tool (NC DETECT). We defined mental health visits as visits with a mental health ICD-9-CM diagnostic code, and organized MHDs into clinically similar groups for analysis.

**Results:**

Those ≥65 with MHDs accounted for 27.3% of all MHD ED visits, and 51.2% were admitted. The most common MHD diagnoses for this age group were psychosis, and stress/anxiety/depression.

**Conclusion:**

Older adults with MHDs account for over one-quarter of ED patients with MHDs, and their numbers will continue to increase as the “boomer” population ages. We must anticipate and prepare for the MHD-related needs of the elderly.

## INTRODUCTION

Older patients with mental health disorders (MHDs) present to the emergency department (ED) with nursing, medical, environmental and social challenges. About 15% of those ≥60 years old have a MHD.^1^ In the US, ED visits by patients ≥65 years old and ED visits by patients with MHDs are increasing.^2^ The World Health Organization has identified the development of age-friendly services and settings as a treatment and care strategy for older adults with MHDs.^1^ Quantifying ED visits by the elderly with MHDs is a first step in improving ED care for this group. Therefore, we analyzed ED visits in North Carolina from 2008–2010 to determine the types and frequencies of ED visits by those ≥65 years old with MHDs, compared with similar ED visits by all other age groups during the same time period.

## METHODS

### Data Collection and Variables

We extracted ED visit data from January 1, 2008-December 31, 2010 from the North Carolina Disease Event Tracking and Epidemiologic Collection Tool (NC DETECT). Over 99% of all civilian ED visits in NC were captured. Details of NC DETECT methodology are available in detail elsewhere.^3^,^4^

A MHD ICD-9-CM diagnostic code in any one of up to 11 positions classified that ED visit as a MHD-related ED visit. Each record was counted as one MHD-related visit if ≥1 MHD diagnosis code was captured for that visit. An MHD diagnosis code was the first listed code in 25% of MHD-related ED visits, and was second- or third-listed in an additional 30% of visits. Of these MHD-related ED visits,79.5% had only one MHD diagnosis code and 16.2% had two MHD codes.

We identified MHDs from ICD-9-CM^5^ codes for Mental Disorders [290–299]: Symptoms, Signs, and Ill-Defined Conditions [787–789.9]; and Supplementary codes [V11-79]. We excluded ICD-9-CM codes for poisoning and overdose, metabolic or structural encephalopathies, which are classified as psychiatric diagnostic codes by ICD-9-CM, tobacco use disorder and substance abuse disorders.

Using the first listed MHD diagnostic code for the ED visit, an expert team of epidemiologists and emergency physicians grouped the MHD diagnoses into 10 clinically similar categories. The following categories were defined using ICD-9-CM codes: stress, anxiety, and depression (300 (excluding 300.9), 306, 308, 309, 311, 313.1, V11.2, V69.8, V79.0); schizophrenia, delusional and paranoid disorders, and psychosis (294.0, 294.8, 294.9, 295, 297, 298, V11.0); bipolar disorder or manic depression (296, V11.1); dementia (290, 294.1, 294.2); suicidal or homicidal ideation (300.9 [often used for homicidal ideation before V62.85 was available], V62.84, V62.85); personality or conduct disorder (301, 312); specific non-psychotic mental disorders due to brain damage (310); pervasive developmental disorders originating in childhood (299); eating disorders (307.1, 307.5); and ‘other’ (302, 307 (excluding 307.1, 307.5, 307.8), V11.8, V11.9, V15.4 (excluding V15.41), V70.1, V70.2, V71.0).^5^

We characterized ED disposition as either admission or discharge from the ED. Admission to a hospital bed or unit (DEEDS codes 110–140) and transfers to another general hospital (DEEDS code 20) were counted as admissions.^6^

### Data Analysis

Data were extracted and stratified for univariate and two-way descriptive analyses. We excluded from this analysis ED records that lacked any diagnosis codes (10.2% of 12,978,615 total ED records). Regression analysis tools implemented in SAS 9.2® were used in multivariable analyses to identify which factors increase the likelihood of hospital admission, after controlling for potential confounders. We calculated descriptive statistics and rates to determine proportions and changes in ED visits over time. Rates were calculated per 10,000 population.^7^ Risk ratios were calculated for hospital admission from the ED. We computed risk ratios using log binomial regression with Poisson robust variances implemented in SAS 9.2 PROC GENMOD. Variables in the model include age (in years: 0–14, 15–24, 25–44, 45–64, ≥65); sex; insurance; day of week; time of day; number of comorbidities; whether the patient visited the ED more than once in the three years studied; year (2008, 2009, 2010); presence of any of 10 co-morbid conditions; and presence of any of 10 psychiatric diagnosis categories (stress, anxiety, and depression; schizophrenia, delusional and paranoid disorders and psychosis; bipolar disorder or manic depression; dementia; suicidal or homicidal ideation; personality or conduct disorder; mental disorders due to brain damage; developmental disorders; eating disorders; and other).

### Study Approvals

The study was approved by the Institutional Review Board of the University of North Carolina School of Medicine and by the Data Use Agreement of the NC Division of Public Health, Epidemiology Division.

## RESULTS

We extracted and analyzed 11,656,207 ED visits from 2008–2010, of which 9.8%, or 1,138,782 visits, had an MHD ICD-9-CM diagnostic code in any position 1–11. Thirty-three percent (33%) of ED visits by all age groups with an MHD diagnostic code were admitted to the hospital, compared to 14% of all ED visits in North Carolina from 2008–2010. The population-based rates of MHD-related ED visits increased progressively from 2008–2010, by 14.4%, while the rate of all NC ED visits increased by only 2.1% during the same time period.^4^

The total proportion of MHD-related ED visits for those age ≥65 years was 27.3% from 2008–2010, and over half of visits (51.2%) resulted in hospital admission, with a relative risk for hospital admission of 2.21, by far the highest proportion for any age group (Table).

The rate of the MHD ‘Dementia’ is highest in ED visits by those aged ≥65 years, an expected finding. However, high ED visit rates for the elderly with the MHD diagnoses ‘Stress/Anxiety/Depressive Disorders’ and ‘Schizophrenia/Delusional Disorders/Psychosis’ were unexpected and exceeded the rate for the MHD ‘Dementia’, and the rates for these three groups of MHDs were far greater in the elderly compared to all other age groups (Table).

## DISCUSSION

In general, studies report that older adults have lower rates of major depressive disorder than younger-aged adults. For example, in one report, the prevalence of major depression was stated as 3.7–4.9% for patients aged 18–64 and only 2.1% for patients 65 and older.^8^ The estimated prevalence of schizophrenia in older adults was also reported to be as low as 0.12% compared to an overall 12-month prevalence of 1.1%.^9^,^10^ Anxiety disorders are reported to affect 3.8% of the elderly population.^1^ Dementia and depression are thought to be the most common mental health [neuropsychiatric] disorders in the elderly.^1^ In contrast, in our study the population-based rates of MHD-related ED visits due to ‘Stress/Anxiety/Depressive Disorders’ and for ‘Schizophrenia/Delusional Disorders/Psychosis’ were actually much higher than for ‘Dementia.’ This finding demonstrates a possible need for systematic ED assessment, and referral for appropriate treatment, for depression and anxiety in older adults. It also emphasizes the need for the appropriate use of non-pharmacologic and pharmacologic modalities to treat agitation and psychosis in those ≥65 in the ED. Furthermore, the high frequency of psychosis in older adults requires that clinicians have the skills to differentiate psychosis from delirium.

Admission to the hospital, especially in older adults with dementia, can be particularly dangerous given the frequency of delirium, falls, and agitation in this population.^11^ Older adults with MHDs may be living longer, or they seek ED care and need hospital admission due to lack of access or resources for care in other ambulatory environments. Because the elderly population is growing, as is the proportion of ED visits for those with MHDs, we can expect that the elderly with MHDs will form an increasing proportion of the ED census. MHD issues in the elderly appear to represent a different pattern and require different approaches than younger patients. Expanded outpatient resources for older adults with MHDs may be needed. If current trends continue in North Carolina, and if other studies or states confirm similar results, then hospitals and EDs must improve the ED and hospital environment for those ≥65 with MHDs, and provide alternatives for ED visits and hospital admissions.

## LIMITATIONS

Diagnostic coding of MHDs is challenging,^12^–^14^ and the authors had no control over individual or institutional coding practices. We have no way of knowing if the order of the diagnosis codes received by NCDETECT is the order in which the clinician, or even the coder, assigned them. That said, we believe that most of the time the first listed diagnosis is probably the primary diagnosis. A previous study comparing the North Carolina Disease Event Tracking and Epidemiologic Collection Tool with the National Hospital Ambulatory Medical Care Survey demonstrated similar rates and proportions of disease groups.^3^

The investigators in this study used face validity in categorizing diagnosis codes into clinically coherent groups. While such groups are somewhat arbitrary, a study reviewing ED visits for MHDs in New South Wales, Australia, using a similar database methodology, resulted in almost identical ICD-9-CM categorization and frequencies of disorders.^15^

NC DETECT captures up to 11 diagnostic codes. In order to capture all relevant MHD codes, especially in the elderly who are likely to have multiple comorbidities, we analyzed all 11 codes. The goal of medical coders is to provide a complete picture of the ED encounter,^16^ and coders do not attach a mental health [psychiatric] code unless it was specifically stated in physician documentation.^17^ Thus, it is reasonable to include a MHD if it was a coded diagnosis in any position, because an MHD can affect the ED differential diagnosis, treatment, or disposition. However, it is possible that many ED visits were not primarily made for mental health issues. We analyzed ED data from only one state, North Carolina. States with different ED mental health or geriatric services could demonstrate different results.

## CONCLUSIONS

The NC population-based rates of ED visits for many MHDs have generally increased from 2008–2010, and the rates of older adult visits mirror this trend.^4^ However, of all age groups, those ≥65 with MHDs accounted for nearly a third of all MHD ED visits, and 51% were admitted. The most common MHDs in the elderly were psychosis and stress/anxiety/depression. Because the elderly population is growing, we anticipate that such ED visits will continue to rise. The needs of the elderly with MHDs must be anticipated, and further research can better predict those needs.

## Figures and Tables

**Table:** The average of rates for years 2008–2010 by age group and category of mental health disorder (MHD) diagnosis/10,000 population, North Carolina; and average proportions of MHD-related emergency department (ED) visits and hospital admissions for years 2008–2010, North Carolina*.

	Age group (years)				
	
Category of MHD diagnoses	0–14	15–24	25–44	45–64	65+
Stress/anxiety/depression	16.2	182.2	285.7	294.0	325.4
Schizo/delusions/psychoses	1.8	19.1	32.8	50.5	337.6
Bipolar	8.5	64.0	93.3	71.6	34.5
Suicidal/homicidal ideation	3.2	20.1	20.8	15.0	4.2
Dementia	0.2	0.3	0.3	3.7	153.9
Personality/conduct disorder	4.2	8.2	5.3	3.8	2.2
Mental disorders d/t brain damage (originating in childhood)	1.1	3.8	2.8	2.0	4.0
Eating disorders	1.2	1.1	0.8	0.4	0.7
Any MHD	47.3	312.1	450.9	448.3	870.5
% of MHD-related ED visits	2.3%	11.0%	31.1%	28.3%	27.3%
MHD-related hospital admission from ED %	14.0%	17.7%	22.2%	36.5%	51.2%
MHD-related relative risk for hospital admission (95% CI)**	1.00 (ref)	1.22 (1.18–1.26)	1.36 (1.31–1.40)	1.79 (1.73–1.86)	2.21 (2.13–2.28)

* Population estimates used as denominators for rate calculations are revised 2008, certified 2009, and projected 2010 estimates obtained 5/16/2011 from Jennifer Song, State demographer, Office of State Budget and Management (North Carolina). These are from North Carolina’s 2010 estimate/projection series and don’t incorporate the 2010 Census counts.

** Risk ratios computed using log binomial regression with Poisson robust variances implemented in SAS 9.2 PROC GENMOD. In addition to categorized number of diagnosis codes (6–11 versus 1–5), variables in model include age (in years: 0–14, 15–24, 25–44, 45–64, 65+); year (2008, 2009, 2010); presence of any of 11 psychiatric diagnosis categories (personality or conduct disorder; dementia; bipolar/manic-depressive; developmental disorder; eating disorder; mental disorder due to brain damage; stress, anxiety, depression; schizophrenic, delusional, psychotic; psychiatric exam or observation; suicidal or homicidal ideation; other mental disorder).
